# Zebrafish Patient-Derived Xenografts Identify Chemo-Response in Pancreatic Ductal Adenocarcinoma Patients

**DOI:** 10.3390/cancers13164131

**Published:** 2021-08-17

**Authors:** Alice Usai, Gregorio Di Franco, Margherita Piccardi, Perla Cateni, Luca Emanuele Pollina, Caterina Vivaldi, Enrico Vasile, Niccola Funel, Matteo Palmeri, Luciana Dente, Alfredo Falcone, Dimitri Giunchi, Alessandro Massolo, Vittoria Raffa, Luca Morelli

**Affiliations:** 1 Department of Biology, University of Pisa, S.S. 12 Abetone e Brennero 4, 56127 Pisa, Italy; a.usai@studenti.unipi.it (A.U.); m.piccardi1@studenti.unipi.it (M.P.); p.cateni@studenti.unipi.it (P.C.); luciana.dente@unipi.it (L.D.); dimitri.giunchi@unipi.it (D.G.); alessandro.massolo@unipi.it (A.M.); vittoria.raffa@unipi.it (V.R.); 2 General Surgery Unit, Department of Translational Research and New Technologies in Medicine and Surgery, University of Pisa, Via Paradisa 2, 56124 Pisa, Italy; gregorio.difranco@med.unipi.it (G.D.F.); matteo.palmeri@med.unipi.it (M.P.); 3 Department of Surgical, Medical, Molecular Pathology and Critical Area, Division of Surgical Pathology, University of Pisa, Via Paradisa 2, 56124 Pisa, Italy; l.pollina@ao-pisa.toscana.it (L.E.P.); niccola.funel@gmail.com (N.F.); 4 Division of Medical Oncology, Pisa University Hospital, Via Roma 67, 56126 Pisa, Italy; caterina.vivaldi@med.unipi.it (C.V.); envasile@tin.it (E.V.); alfredo.falcone@med.unipi.it (A.F.)

**Keywords:** zebrafish avatar, chemosensitivity, preclinical model, pancreatic cancer, personalized medicine

## Abstract

**Simple Summary:**

Treating the PDAC (pancreatic ductal adenocarcinoma) zPDXs (zebrafish patient-derived xenografts) with chemotherapy regimens commonly used, we performed a co-clinical trial testing the predictiveness of the model. We found that zPDX may predict patient outcomes, classifying them into responders (R) and non-responders (NR), reporting a statistically significant higher cancer recurrence rate at 1 year after surgery in the NR group: 66.7 versus 14.3%. Our zPDX model seems to be a promising tool for the stratification of PDAC patients. This is a crucial starting point for future study involving more patients to obtain a method to really personalize the oncological treatment of PDAC patients.

**Abstract:**

It is increasingly evident the necessity of new predictive tools for the treatment of pancreatic ductal adenocarcinoma in a personalized manner. We present a co-clinical trial testing the predictiveness of zPDX (zebrafish patient-derived xenograft) for assessing if patients could benefit from a therapeutic strategy (ClinicalTrials.gov: XenoZ, NCT03668418). zPDX are generated xenografting tumor tissues in zebrafish embryos. zPDX were exposed to chemotherapy regimens commonly used. We considered a zPDX a responder (R) when a decrease ≥50% in the relative tumor area was reported; otherwise, we considered them a non-responder (NR). Patients were classified as Responder if their own zPDX was classified as an R for the chemotherapy scheme she/he received an adjuvant treatment; otherwise, we considered them a Non-Responder. We compared the cancer recurrence rate at 1 year after surgery and the disease-free survival (DFS) of patients of both groups. We reported a statistically significant higher recurrence rate in the Non-Responder group: 66.7% vs. 14.3% (*p* = 0.036), anticipating relapse/no relapse within 1 year after surgery in 12/16 patients. The mean DFS was longer in the R-group than the NR-group, even if not statistically significant: 19.2 months vs. 12.7 months, (*p* = 0.123). The proposed strategy could potentially improve preclinical evaluation of treatment modalities and may enable prospective therapeutic selection in everyday clinical practice.

## 1. Introduction

Pancreatic ductal adenocarcinoma (PDAC) is a high-malignancy disease with rapid progression and poor prognosis. PDAC originates in the exocrine pancreas and it accounts for more than 90% of all pancreatic neoplasms [1]. In 2021, it is estimated that pancreatic cancer will be the third-leading cause of cancer death worldwide, with a 5-year overall survival rate lower than 5% and a median survival time of 7–11 months [2]. The incidence of PDAC has been steadily rising since 2004 in European countries, as well as in the U.S., and it is projected to nearly double the number of cases by 2030, becoming the second-most-common cause of cancer-related deaths [3]. The poor prognosis is due to the limited predictive biomarkers for early detection [4].

Neoadjuvant or adjuvant cytotoxic chemotherapy in association with PDAC resection represents the current standard of care, but the benefits in term of survival are often modest [5]. Overall, clinical studies have shown that many PDAC patients have chemorefractory disease, and a significant response to chemotherapy is exhibited just by a small subgroup [6].

In this scenario, the challenge is to discover and develop novel safe and more effective therapies, and, at the same time, the necessity of new predictive tools to test the clinical performance of therapies to improve survival and quality of life for patients is increasingly evident [7].

Nowadays, the promising PDAC preclinical models are the patient-derived organoids (PDO), such as an in vitro system wherein progenitor cells are cultured in 3 dimensions (3D) with the possibility of reconstituting niches more similar to PDAC [8,9] or in vivo patient-derived xenografts (PDXs) with zebrafish (zPDX, *Danio rerio*) or mouse (mPDX, *Mus musculus*) as recipients [10,11]. PDXs are developed by implanting patient tumor tissue or primary cells into the animal models, recapitulating and maintaining the main features of the original tumor, and they can be used in drug efficacy studies [12,13,14,15,16,17]. Numerous studies have highlighted the application of PDO and PDX in the emerging field of personalized cancer medicine, and it is commonly accepted that these clinically relevant preclinical models could be crucial in accurately predicting patient response in clinical trials [18,19]. However, the generation of mPDX requires a large amount of tissue, and they take months to establish [20], as well as the PDO that need long experimental time to reach a stable culture [21].

Therefore, in this study, we present the outcome of the first zebrafish larval co-clinical trial [22] conducted by our research group [10,23], adding a comparison of the tests performed on zPDXs with clinical data on responses to adjuvant chemotherapy, with the aim of addressing the question: “Will a specific patient benefit from a chemotherapy regimen?”. Compared to the well-consolidated procedure of isolated cancer-cell xenografts, we adopted an approach consisting of the xenotransplantation of pieces of the patient tumor tissue into zebrafish embryos, which have not yet developed an adaptive immune response (XenoZ, NCT03668418). Treating the PDAC zPDXs with chemotherapy regimens commonly used in clinics, we tested the predictiveness of the model for assessing patient profiles in terms of being a responder/non-responder to chemotherapy, which may have implications for making clinical decisions in everyday clinical practice.

## 2. Results

### 2.1. Clinical Data of PDAC Patients

From July 2018 to June 2020, a total of 31 patients with PDAC were enrolled (Table 1).

Of these, 18 (58.1%) were male. The mean age was 71.8 ± 8.2 years (range 44–85), and the mean BMI was 25.5 ± 4.4 (range 17.6–40.4). A pancreatoduodenectomy was performed in 23/31 (74.2%) patients, a distal splenopancreatectomy in 7/31 patients (22.6%) and a total splenopancreatectomy in 1/31 (3.2%) patients. An associated vascular resection was performed in 3/31 (9.7%) patients: a venous resection in two cases and a resection of the celiac trunk in one case. No intra-operative complications were reported, and, in all cases, it was possible to take a fragment of the tumor from the surgical specimen for xenotransplantation in zebrafish embryos. The histological examination confirmed the presence of a PDAC in all cases. A moderately differentiated adenocarcinoma (G2/3) was reported in 23/31 (74.2%) cases, while a poorly differentiated adenocarcinoma (G3/3) was reported in 8/31 (25.8%) cases. The mean diameter of the pancreatic neoplasia was 3.2 ± 1.0 cm (range 1.5–5.0). The mean number of harvested lymph nodes was 39.5 ± 16.6 (range 17–74), while the mean number of positive lymph nodes was 5.2 ± 4.7 (range 0–22). The presence of positive lymph nodes was documented in 29/31 (93.5%) patients: a N1 status was reported in 12/31 (38.7%) patients, while a N2 status was reported in 17/31 (54.8%) patients. The presence of perineural infiltration was reported in 24/31 (77.4%) patients, while angioinvasion was reported in 3/31 (9.7%) patients. Histological examination confirmed the presence of vascular infiltration in 2/3 cases of vascular resection.

### 2.2. Zebrafish Trial

**Figure 1:**
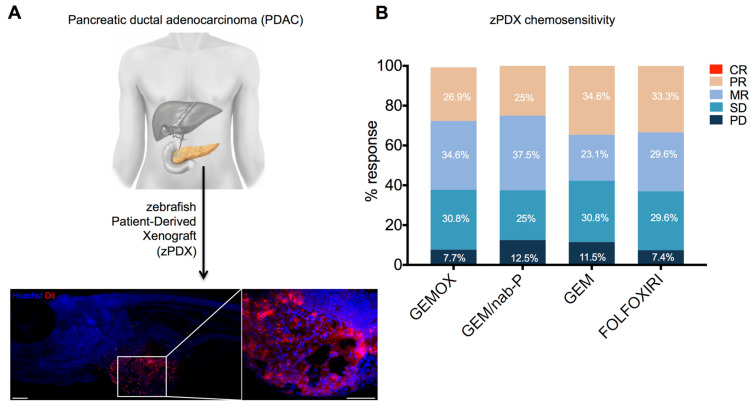
Zebrafish patient-derived xenograft model and type of response to chemotherapy drugs. (**A**) zPDXs were established from the transplantation of fresh PDAC human tissue (DiI-labeled) into the perivitelline space of 2 dpf zebrafish embryos. zPDXs were then treated with GEMOX, GEM/nab-P, GEM and FOLFOXIRI for two days to detect their chemosensitivity profile. Representative image on the bottom (the right image is the magnification of the white-delimited area). Scale bars = 50 µm. (**B**) Percentage of progressive disease (PD), stable disease (SD), minor response (MR), partial response (PR) and complete response (CR). No statistically significant differences were detected by a Chi-square test. (*n* = 26, 24, 26, 27 zPDXs, respectively, for GEMOX, GEM/nab-P, GEM and FOLFOXIRI).

We obtained 31 human samples from PDAC primary tumors isolated from surgical resections and we successfully generated 27 zPDXs (efficiency of 87%) (Figure 1A).

For the zPDXs of PDAC, we observed a progressive disease (PD) in 7.4%, 7.7%, 11.5% and 12.5% of cases, respectively, with FOLFOXIRI, GEMOX, GEM and GEM/nab-P. Furthermore, 25% and 29.5% of SD was observed, respectively, with GEM/nab-P and FOLFOXIRI, and 30.8% of SD both for GEMOX and GEM. MR was detected in 23.1%, 29.6%, 34.6% and 37.5%, respectively, with GEM, FOLFOXIRI, GEMOX and GEM/nab-P. Furthermore, 34.6% PR with GEM and 33.3% with FOLFOXIRI, 26.9% PR with GEMOX and 25% with GEM/nab-P were all observed. No CR was observed for any of the chemotherapy treatments (Figure 1B). No statistically difference was detected (*p* = 0.964).

### 2.3. Data Modelling

**Figure 2:**
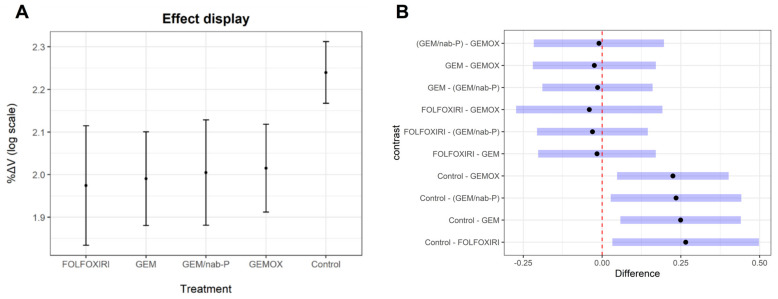
Effects of chemotherapy drugs in the linear mixed effect model. (**A**) Effect displays. The treatments are displayed on the x-axis. Dots identify the fixed effect values of %ΔV estimated by LMM. The bars are the 95% CI of fixed values. (**B**) Post-hoc test results. Pairwise comparisons are on the y-axis, and the differences of marginal means between treatments are on the x-axis. Blue bars represent the 95% CI of means differences. The dashed line is a difference equal to zero between means.

LMM showed that, compared to controls, all the treatments induced a significant reduction of tumor mass (Figure 2A, Table S1).

The post hoc tests indicated that all the proposed treatments caused a regression of the tumor volume, but no results were significantly different from the others (Figure 2B).

Regarding the zPDX tumor behaviors with respect to population mean, we observed a significant reduction of tumor mass with FOLFOXIRI in 3 out of 20 zPDXs (15%) and in 4 out of 20 zPDXs (20%) both in GEM and GEM/nab-P, whereas GEMOX did not show any significant reduction of volume (Figure S1).

**Figure 3:**
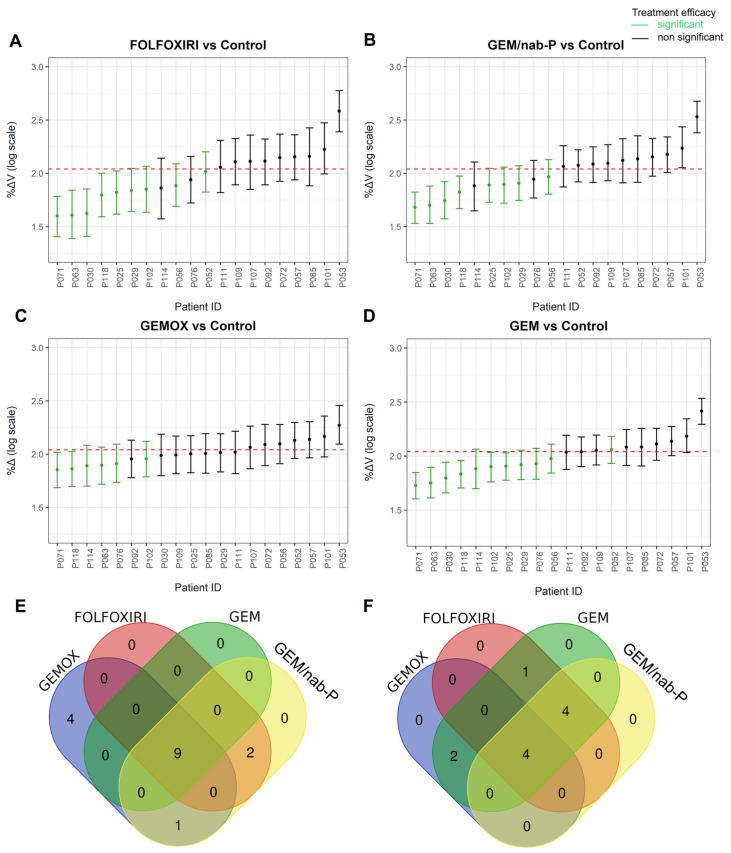
Analysis of LMM predicted %ΔV and 95% CI for each chemotherapy scheme. Predicted values of %ΔV and 95% CI in (**A**) FOLFOXIRI, (**B**) GEM/nab-P, (**C**) GEMOX and (**D**) GEM. These values were obtained by adding the LMM fitted values of fixed effects to random values and to their simulated 95% CI. The dashed line is 0 on the log scale. The 95% CI above and intersecting the line identify zPDXs with a non-significant reduction of tumor volume. The 95% CI below and not intersecting the line are zPDXs with a significant reduction of tumor volume. Green bars are zPDXs in which tumor mass is significantly reduced compared to control. To determine the significant reduction of tumor volume with respect to control, we overlapped the 95% CI of treatments and control groups. Patient enrollment codes are reported (P = pancreas). (**E**) Intersection sets of zPDX classified as “non-significant” and (**F**) “significant” in Figure 3 (table data with the list of patient codes is provided as Table S1).

Analysis of predicted %ΔV and 95% CI showed that FOLFOXIRI was statistically significant efficient in 9/20 cases (45%) with respect to the control group, and in 5/20 cases (25%) with respect to 0 on the log scale (Figure 3A). GEM/nab-P treatment was statistically significant effective in 8/20 zPDXs (40%) with respect to controls, and in 4/20 zPDXs (20%) with respect to 0 on the log scale (Figure 3B). GEMOX was observed significantly efficient in reducing tumor volume with respect to the control group in 6/20 zPDXs (30%) and in 2/20 zPDXs (10%) with respect to 0 on the log scale (Figure 3C). zPDXs treated with GEM registered a significant reduction of tumor volume in 11/20 cases (55%) with respect to the control group, and in 6/20 cases (30%) with respect to 0 on the log scale (Figure 3D). Intersecting the zPDXs that responded non-significantly with the types of chemotherapy, it was possible to observe how zPDXs that did not respond significantly did so for all four schemes in 9/16 (56.3%) or for most of the schemes in 3/16 (18.8%) (Figure 3E). On the other hand, it was possible to observe how zPDXs that responded significantly did so for either all four schemes in 4/11 (36.4%) or for three schemes in 4/11 (36.4%) (Figure 3F). The predicted values for each zPDX are reported in Figure S2.

### 2.4. Comparison of zPDX Drug Treatment with Short-Term Patient Treatment Responses

**Figure 4:**
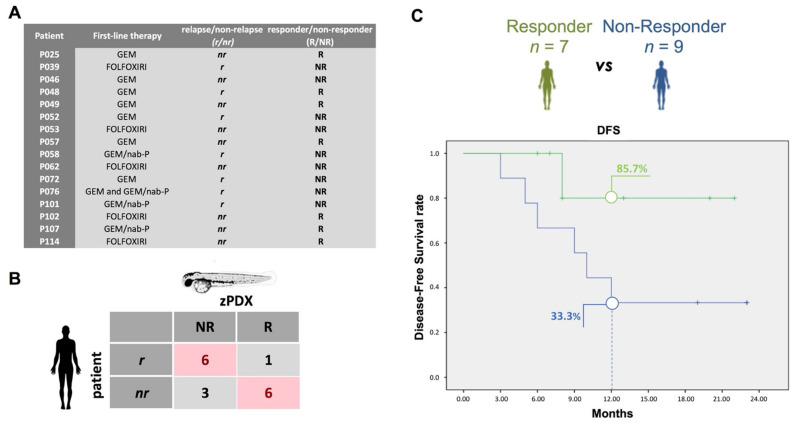
Analysis of the follow-up data in comparison to the prediction of the zPDX. (**A**) Relapse/non-relapse (r/nr) information for 16 PDAC patients enrolled in Table 2 versus the respective responder/non-responder (R/NR) zPDXs. We considered relapse when the patient has the clinical evidence of recurrence within one year after surgery. The zPDX treatment response may predict an early relapse (r) or a better response to therapy (non-relapse, nr). Sixteen PDAC zPDX, corresponding to patients subjected to curative surgery and postoperative adjuvant treatment, were treated with GEM, GEM/nab-P, GEMOX and FOLFOXIRI for 2 days. The zPDX response to treatment was analyzed and quantified adapting the WHO criteria for tumor response. We considered responder (R) zPDX with a decrease ≥50% in the relative tumor area. (**B**) Confusion matrix highlights the number of no cancer relapse (nr) in patients with the own responder (R) zPDX and the number of cancer relapse (r) in patients with the own non-responder (NR) zPDX. (**C**) Disease-free survival difference in R group (green) and NR group (blue), *p* = 0.123 by log-rank test.

Of the 31 patients with PDAC enrolled, 7 patients did not receive adjuvant chemotherapy, either as a choice of the patients or due to poor recovery after surgery; 2 patients died after the surgical operation; 3 patients received an adjuvant chemotherapy scheme not tested in zPDX; in 2 cases, we reported a high mortality rate of the zPDX or tumor tissue that did not engraft in zebrafish embryos, and one patient dropped out of the co-clinical trial. For 16 cases, we had both oncological information obtained during follow-up, in term of relapse/non relapse (Figure 4A), and the zPDX chemosensitivity profile (Figure 4B).

The median follow-up was 19.5 months (range 5–23 months). Seven of these patients (43.8%) had a cancer recurrence during the follow-up, and all of these recurrences occurred during the first year after surgery (Figure 4A) with a median disease-free survival (DFS) of 12 months.

Of the sixteen patients, we observed a responder zPDX with the same chemotherapy scheme used as adjuvant treatment in 7 (43.75%) cases (Responder-group, R). At one year after surgery, the patients of the R-group reported cancer recurrence in only 1/7 patient (14.3%), while the patients of the NR-group (NR) reported cancer recurrence in 6/9 cases (66.7%) (Figure 4C, p = 0.036 by a Chi-square test). No statistical differences were detected in the clinical data between the R-group and the NR-group (Table 2); also, no differences were found in terms of the type of adjuvant chemotherapy schemes and mean follow-up between the two groups.

The mean DFS was longer in the R-group with respect to the NR-group, even if not statistically significant: 19.2 months vs. 12.7 months, *p* = 0.123 by a log-rank test (Figure 4C). The relative risk of recurrence is estimated to be RR = 0.21.

## 3. Discussion

PDAC is a highly lethal malignancy with a 5-year survival rate of 5% [2]. To date, the only potentially curative option is surgery in combination with chemotherapy [24]. Nevertheless, early recurrence and disease progression after surgery are evident in a large proportion of patients [25,26]. Improvement of the chemotherapy treatment options was obtained with the introduction of combination therapies over single-agent gemcitabine. However, better oncological outcomes, such as increased overall survival reported with FOLFOXIRI and GEM/nab-P compared to only-gemcitabine, are associated with increased incidence of adverse events [27,28]. Since effective therapeutic strategies for patients with PDAC have been difficult to identify [29], proper patient selection could be crucial to identify who may take advantage from an aggressive chemotherapy approach [30]. With this intent, the concept of personalized medicine has emerged in recent years with the aim of tailoring medical treatment to the individual characteristics of each patient, and particularly, to the tumor biology of each patient [31]. Different patient-derived tumor models, both in vitro and in vivo, have been developed, each with its own peculiarities [32,33]. The use of zebrafish embryos has several advantages with also the possibility of overcoming some drawbacks of murine xenografts, such as the larger number of tumor cells needed (about 1 million), the long time required (from several weeks to months) to have a visible tumor implant, the need of immunosuppressed animals to avoid transplant rejection and the high difficulties of generating mouse xenotransplant models able to metastasize. Moreover, the aquatic environment of the zebrafish allows one to directly dissolve the drugs in the embryo water, avoiding the burden of administering the drug to each individual animal [34,35]. With this intent, our first step was to establish and validate the equivalent of a human dose for fish that was effective both for cancer cell lines and for tumor tissue xenotransplanted in zebrafish embryos [23]. The results were very promising despite the short drug exposure and the non-physiological temperature of both the zebrafish embryos (28 °C) and the tumor cells (37 °C), which do not affect the cell engraftment, in line with the evidence collected by the previous literature [36].

The strategy that we developed in our zPDX model has the distinct advantage of preserving the tumor-associated stroma and the microenvironmental factors, maintaining the original tumor architecture and the histological characteristics, as we reported in our previous article, in which, at histological examination, we observed the presence of both epithelial cells and stromal cells, with a percentage of epithelial cells (mean PDAC counterpart) out of the total surface area similar to that of the pancreatic tumor tissue [10].

On the other hand, the lack of enzymatic digestion, adopted to retain the extracellular matrix composition and the three-dimensional structure of cancer tissues, could implicate the limitation of tumor heterogeneity being less represented in a tissue fragment than in a cellular suspension. Indeed, small tissue pieces could present high differences between each other’s in terms of benign cell populations and tumor subclones. For that reason, we proposed to overcome this criticism by increasing the number of xenografted embryos. This could also tackle the problem of the lower survival rate of our zPDX model, ascribed to the invasiveness of the transplantation technique, which is more traumatic for embryos compared to the injection of a cell suspension with microcapillaries.

It is well known that the microenvironment and tumor heterogeneity influence the response to chemotherapy treatment. Specifically, in pancreatic cancer, the microenvironment consists mostly of cancer-associated fibroblasts, immune cells, the extracellular matrix and many other secondary elements [37] that create a dense stromal fibrosis with the consequence of generating a considerable obstacle to therapeutic intervention [38]. In this context, our zPDX, which maintains the human tumor’s microenvironment, makes treating PDAC more realistic because therapies target not only the cancer cells themselves but also the stroma [39,40].

Until now, very few studies have evaluated the possibility of xenotransplant patient-derived tumor cells taken from the surgical specimen directly in zebrafish embryos to create an avatar for oncological patients with the intent to predict the type of response to adjuvant chemotherapy for solid tumors. Moreover, all of them have the limitation of a small number of patients enrolled, and none of them involved patients with pancreatic cancer. Wu et al. [14] showed a retrospective correlation with one gastric tumor patient’s clinical outcome, while Fior et al. [41], in a retrospective study, observed that colorectal patient’s avatars were predictive of patient clinical outcome in 4 out 5 patients (80%). Our study is a prospective study involving 31 PDAC patients, of whom we successfully generated 27 zPDXs with an efficiency of 87%, which is currently in line with a success rate >80% of a different preclinical model, such as PDAC organoid establishment [8,42].

From 27 zPDXs, 16 were usable for the co-clinical trial to compare the zPDX chemosensitivity profile and the clinical response to adjuvant chemotherapy.

In our preliminary experience recently published, we reported very encouraging preliminary results [10]. First, we observed the possibility of directly xenotransplanting tissue taken from PDAC in zebrafish embryos, obtaining in all cases of the control group an increase of the relative tumor area (2 dpi/1 dpi). Moreover, comparing the results of zPDX tests with data on chemoefficacy published in literature, the model seemed to reflect the different efficacy of the various chemotherapy schemes used for the treatment of patients affected by PDAC [10].

To have another line of evidence, in the current study, we performed a stratification analysis of the zPDXs, classifying them as significant and non-significant, according to the LMM analysis plotted in Figure 3. Data point out that zPDXs had a strong tendency to share the same pattern of response to treatments (Figure 3F), suggesting that zPDX models could be a preclinical platform for the assessment of drug efficacy, identifying a group of patients that are more likely to benefit from treatment.

The further crucial step of this analysis was the association of experimental data with clinical data obtained during the follow-up of the enrolled patients who had undergone adjuvant chemotherapy. To do that, we performed a proof-of-concept analysis of the co-clinical study to test our zPDX model as a platform to study response to treatment.

For 16 patients enrolled in the co-clinical trial, we tested whether response to treatment in zPDX (responder—R) would predict a delay in relapse in the matching patients (non relapse—nr), or whether resistance to drug treatment in our zPDX model (non-responder—NR) would associate with an early tumor relapse (r). In this way we obtained two groups of patients, the responder-group and the non-responder-group, of whom the first one theoretically should be associated with better oncological outcomes. The two groups did not differ in term of both histological characteristics of the tumor and mean length of follow-up. Therefore, because all of them underwent complete surgical resection of PDAC, we evaluated the response to adjuvant chemotherapy using both the cancer recurrence rate and the DFS, in accordance with the methods used by oncologists to compare the efficacy of different chemotherapy schemes in patients who had previously undergone the complete surgical resection of the neoplasm [43,44,45]. Interestingly, we reported a statistically significant higher cancer recurrence rate in the non-responder-group with a cancer recurrence in 66.7% of cases in contrast to 14.3% of cases in the responder-group. Moreover, the mean DFS results were longer in the responder-group than the non-responder-group, being respectively 19.2 months vs. 12.7 months, even if it was not statistically significant, probably due to the small sample size (Table 2, Figure 4). Thus, we could anticipate relapse/no relapse within 1 year after surgery in 12 out of 16 patients.

These results were very encouraging. In fact, we observed that our zPDX model seems to identify PDAC patients who are more likely to respond to chemotherapeutics and who are associated with favorable survival odds. This is a crucial point for the personalized medicine of PDAC patients. In fact, many PDAC patients have chemo-refractory disease, and a smaller subset exhibits significant response to chemotherapy. To date, some PDAC mutations have been individuated, such as microsatellite instability, BRCA2 mutations and potentially targetable, uncommon KRASG12C mutations that influence the chemotherapy response [8]. However, there are a considerable number of patients without these genetic alterations that would benefit from alternative treatment strategies because they will probably not respond to the current chemotherapy. However, current therapeutic selection for both local and metastatic pancreatic cancer patients is often based on patient performance status and co-morbidities. Overall, this highlights the unmet clinical need to define subgroups of chemotherapy-responsive patients to guide treatment selection and to decide alternative treatment options for patients who are resistant to currently approved treatment regimens. Therefore, the chemo-sensitivity definition of the PDAC of each patient may enable more rapid treatment stratification of PDAC patients into those who may benefit from currently available chemotherapeutic interventions and those who should instead be considered for investigational agents.

## 4. Materials and Methods

### 4.1. Human Trial

The observational prospective co-clinical trial (ClinicalTrials.gov: XenoZ, NCT03668418, study started on June 1, 2018) was conducted at the University of Pisa (Pisa, Italy) in accordance with the guidelines of the European Network of Research Ethics Committees. The local ethics committee on clinical testing approved the study (prot. n. 70213). All the donors enrolled in the study provided signed informed consent. The main inclusion criteria were 18 years-of-age or older patients diagnosed with PDAC who had not previously been treated with chemotherapy. The exclusion criteria were significant co-morbid cardiovascular and respiratory disease, history of prior cancer or prior treatment with any chemotherapy regimen, pregnant or lactating females, life expectancy <12 weeks and patients requiring urgent/emergency interventions.

Human samples were obtained from primary tumors surgically resected. A pathologist analyzed the specimen, and a fragment of the tumor was taken for xenotransplantation in zebrafish embryos.

Preoperative data included diagnosis, age, gender, body mass index (BMI), value of tumor marker Ca 19.9 and neoadjuvant therapy. Operative data included type of surgical procedure, if an associated vascular resection was performed and if there were problems in taking a fragment of the tumor for the xenotransplantation. Histological data included: histological type of the tumor, grade of differentiation, tumor dimension, number of harvested lymph nodes, number of metastatic lymph nodes, presence of angioinvasion and perineural infiltration and the presence of vascular infiltration in the case of vascular resection. Patients were staged according to the T and N definitions proposed for the American Joint Committee on Cancer 8th edition [46]. The proposed T-stage definitions are the following: T1 ≤ 2 cm maximal diameter, 2 < T2 ≤ 4 cm maximal diameter, T3 > 4 cm maximal diameter, T4 = locally unresectable. Extra-pancreatic extension was not included in these T-stage definitions. Proposed N-stage definitions included the following: N0 = node negative, N1 = 1–3 nodes positive for metastatic disease, N2 ≥ 4 nodes positive for metastatic disease. The follow-up data included the administration or not of adjuvant chemotherapy and the type of the chemotherapy scheme, the cancer recurrence and the DFS. DFS was defined as the time from pancreatic surgical resection to cancer recurrence or death from any cause, whichever occurred first. After surgery, all the included patients underwent radiological assessment as per clinical practice. The patients were classified as relapse/no relapse (r/nr) if they relapsed or did not relapse.

### 4.2. Zebrafish Welfare Assurance and Husbandry

Zebrafish were handled in accordance with local animal welfare regulations (authorization n. 99/2012-A, 19 April 2012; authorization for zebrafish breeding for scientific purposes released by the “Comune di Pisa” DN-16/43, 19 January 2015) and the procedures were approved by Italian Ministry of Public Health, in conformity with the Directive 2010/63/EU. The zebrafish used in this study were kept at 37 °C in tanks housed on a custom-built, stand-alone, re-circulating system (Tecniplast, Varese, Italy). Zebrafish fertilized eggs were obtained by natural mating of AB wild-type fish at our facilities and the developing embryos were staged in an incubator at 28 °C, according to Kimmel et al. [47]. Before any procedure, embryos were anesthetized in 0.02% tricaine.

### 4.3. Human Material for Zebrafish Xenografts

Detailed procedures for tissue transplantation have been previously described by Usai et al. [23]. Briefly, bulk tumor tissue screened by the histopathologist (at the Division of Surgical Pathology, University of Pisa) was washed three times with RPMI supplemented with 100 U/mL penicillin, 100 µg/mL streptomycin and 2.5 µg/mL amphotericin; then, it was minced, firstly with a scalp blade (1–3 mm), and then using the McIlwain tissue chopper (Campden Instruments LTD, Loughborough, UK) to obtain pieces of about 0.3 mm × 0.3 mm × 0.3 mm. The pieces were stained with 10 µg/mL CM-Dil (Invitrogen, Carlsbad, CA, USA) in D-PBS and incubated for 30 min at 37 °C. Tissue pieces were then washed and centrifuged three times by D-PBS and resuspended in D-PBS supplemented with 10% FBS (Gibco, Waltham, MA, USA). Pieces of fluorescent-labeled tissue were manually transplanted into the perivitelline space of *n* = 90 AB wild-type recipient embryos 2 days post fertilization (dpf), which were lying in 1% agarose disks in multi-well plates. After transplantation, embryos were incubated for 2 h at 35 °C. The pool of embryos xenografted with the tissue derived from each patient will be hereinafter referred to as zPDXs.

### 4.4. Assessing Therapeutic Responses in Zebrafish Xenografts

Embryos, previously selected for the presence of tissue, were randomly and equally allocated among 5 groups (4 therapeutic options and 1 control group, *n* ≥ 10 of xenografted embryos/group) and imaged at 2, 24 and 48 h post injection. After each time point, fresh drugs were administered at the equivalent dose (ED = 5) validated in our previous study [23], and embryos were incubated at 35 °C. The chemotherapy schemes tested were gemcitabine (GEM), gemcitabine + oxaliplatin (GEMOX), gemcitabine + nab-paclitaxel (GEM/nab-P), and 5-fluorouracil + folinic acid + oxaliplatin + irinotecan (FOLFOXIRI) (Table 3). Control embryos were maintained in E3 supplemented with 100 U/mL penicillin and 100 µg/mL streptomycin.

The effects of chemotherapy on zPDXs were firstly assessed by adapting the WHO criteria based on bidimensional measurements [48] and classifying the outcomes into 5 groups comparing the response to chemotherapy to the relative stained area of the control group at 2 days post injection (dpi): Complete response (CR), partial response (PR), minor response (MR), stable disease (SD) and progression disease (PD) (Table 4).

### 4.5. Co-Clinical Trial

Clinical data obtained during the follow-up were compared to data of the therapeutic response in zPDX. Patients who died during the hospital stay after surgical operation, who did not undergo adjuvant chemotherapy or who underwent chemotherapy schemes not tested in xenografted zebrafish embryo, were excluded from the analysis. Moreover, patients whose zebrafish avatar reported low engraftment were excluded from the analysis (*n* ≥ 3 embryos for each group). We considered zPDX a responder (R) when a decrease ≥ 50% in the relative tumor area was reported; otherwise, they were considered a non-responder (NR). We further classified patients according to the outcome of their zPDX: the patient was considered a Responder if her/his own zPDX was classified as a R for the chemotherapy scheme she/he received as adjuvant treatment; otherwise, she/he was classified as a non-responder. Our endpoint was to compare the recurrence rate at 1 year after surgery of the patients of the responder-group and non-responder group. The disease recurrence rate and the DFS of the two groups were compared using the log-rank test. The relative risk (RR) was calculated as the ratio of the probability of recurrence in the responder group to the probability of recurrence in the non-responder group.

### 4.6. Linear Mixed Effect Model Data Analyses

To maximize the prediction of tumor variation, we developed a method for modeling how it changes in three dimensions. Thus, we estimated the volume at 1 dpi and 2 dpi assuming that the tumor volume could be approximated as a sphere and considering area as a circular section of the mass.

We got volume variation as:

$$\%\Delta V=\frac{V_{2\mathrm{dpi}}-V_{1\mathrm{dpi}}}{V_{1\mathrm{dpi}}}\;\times \;100$$

We used percent change in tumor volume (%Δ*V*) as a proxy to evaluate treatment efficacy on PDAC zPDXs.

Due to the hierarchical structure of data, we opted for a linear mixed effect model (LMM) [49]. All statistical analyses were performed using R software version 4.0.4. (https://www.r-project.org/, accessed on 15 February 2021).

Treatments (5 classes) were considered fixed effects, whereas zPDXs were the random effects (20 categories). Fixed effects indicated the mean effect of each treatment type on zebrafish embryo population. Random effects evaluated each zPDX response to proposed treatments with respect to the population mean.

The model also considered random components as intercept and slope [49]. The analysis of random slope allows us to examine zPDX-specific responses to treatments. The proposed model is the result of a selection process: as the first step we started with an LMM consisted of random components of the intercept, then, according to our purpose, we made an LMM comprising both random intercept and slope.

In our design, treatment was a nominal variable with five categories (four treatment options plus a control group used as a baseline). The LMM outcome variable was the %ΔV that showed a positively skewed distribution, so we decided to transform this variable to satisfy linear regression assumptions [50].

We added 110 to %Δ*V*, a constant value that allowed us to apply 10-base logarithm transformation. %Δ*V* was transformed as:

$$\%\Delta V=log_{10}\left(\%\Delta V_{untransformed}+110\right)$$

Using the fixed effect coefficients estimated through the LMM, a post hoc test was performed with the R package emmeans. We calculated marginal means for each dummy variable, their standard errors and 95% confidence intervals (95% CI). We computed pairwise comparisons of least square means with adjusted p-values by Tukey’s HSD method.

To evaluate if there was a statistically significant dissimilarity in zPDX response to treatments compared to the overall population, we simulated 95% CI of random effects for each treatment and contrasted them to the population mean, represented by fixed effect coefficients.

Additionally, to identify treatment efficacy with respect to the control group, for each zPDX, we contrasted 95% CI of predicted values in treatments and in the control group. To obtain predicted values, we added the fitted values of fixed effects to random values and to their simulated 95% CI. To assess the significant reduction of tumor volume with respect to control, we overlapped the 95% CI of treatments and control groups. Furthermore, we tested the significant tumor volume reduction with respect to 0 on the log scale using the 95% CI of predicted %Δ*V*. When the 95% CI of predicted %Δ*V* was less than 0 on the log scale, the tumor volume of the corresponding zPDX is significantly reduced.

## 5. Conclusions

In conclusion, our zPDX model seems to be a promising tool for the stratification of PDAC patients based on their theoretical response to current chemotherapy schemes. This is a crucial starting point for future study involving more patients to obtain a method to really personalize the oncological treatment of PDAC patients.

## Figures and Tables

**Table 1:** Characteristics of the PDAC patients enrolled (*n* = 31).

Characteristics	
Mean age, years ± SD	71.8 ± 8.2 (44–85)
M:F, *n* (%)	18:13 (58.1%)
Mean BMI, kg/m^2^ ± SD	25.5 ± 4.4 (17.6–40.4)
Type of surgical procedure, *n* (%)	
Pancreatoduodenectomy	23 (74.2%)
Distal splenopancreatectomy	7 (22.6%)
Total splenopancreatectomy	1 (3.2%)
Vascular resection, *n* (%)	3 (9.7%)
Grade of differentiation, *n* (%)	
G2/3	23 (74.2%)
G3/3	8 (25.8%)
Mean tumor dimension, cm	3.2 ± 1.0 (1.5–5.0)
Mean harvest lymph nodes, *n*	39.5 ± 16.6 (17–74)
Mean positive lymph nodes, *n*	5.2 ± 4.7 (0–22)
T status, *n* (%)	
T1	3 (9.7%)
T2	19 (63.3%)
T3	9 (30.0%)
N status, *n* (%)	
N0	2 (6.5%)
N1	12 (38.7%)
N2	17 (54.8%)
Stage, *n* (%)	
IA	1 (3.2%)
IB	1 (3.2%)
IIB	12 (38.7%)
III	16 (51.6%)
IV	1 (3.2%)
Angioinvasion, *n* (%)	3 (9.7%)
Perineural infiltration, *n* (%)	24 (77.4%)
Vascular infiltration, *n* (%)	2 (6.7%)

BMI: body mass index; M: male; F: female.

**Table 2:** Clinical and biological characteristics of PDAC patients eligible for the follow-up (*n* = 16).

Characteristics	Responder (*n* = 7)	Non-Responder (*n* = 9)	*p* Value
Mean age, years ± SD	71.4 ± 5.3	67.9 ± 11.0)	0.448
M:F, *n* (%)	2:5 (28.6%:71.4%)	7:2 (77.8%:22.2%)	0.049
Mean BMI, kg/m^2^ ± SD	26.3 ± 6.7	24.4 ± 3.1	0.448
Type of surgical procedure, *n* (%)			0.091
Pancreatoduodenectomy	6 (85.7%)	4 (44.4%)	
Distal splenopancreatectomy	1 (14.3%)	5 (55.6%)	
Vascular resection, *n* (%)	0	1 (11.1%)	0.362
Grade of differentiation, *n* (%)			0.042
G2/3	7 (100%)	5 (55.6%)	
G3/3	0	4 (44.4%)	
Mean tumor dimension, cm	2.9 ± 0.5	3.4 ± 1.1	0.209
Mean harvest lymph nodes, *n*	41.1 ± 21.6	38.7 ± 18.8	0.810
Mean positive lymph nodes, *n*	5.0 ± 3.6	5.4 ± 4.0	0.820
T status, *n* (%)			0.059
T1	0	1 (11.1%)	
T2	7 (100%)	4 (44.4%)	
T3	0	4 (44.4%)	
N status, *n* (%)			0.949
N1	3 (42.9%)	4 (44.4%)	
N2	4 (57.1%)	5 (55.6%)	
Stage, *n* (%)			0.635
IIB	3 (42.9%)	4 (44.4%)	
III	4 (57.1%)	4 (44.4%)	
IV	0	1 (11.1%)	
Angioinvasion, *n* (%)	0	1 (11.1%)	0.362
Perineural infiltration, *n* (%)	5 (71.4%)	6 (66.7%)	0.838
Vascular infiltration, *n* (%)	0	0	1
Type of adjuvant chemotherapy schemes, *n* (%)			0.574
Gemcitabine	4 (57.1%)	3 (33.3%)	
GEM/nab-P	1 (14.3%)	3 (33.3%)	
FOLFOXIRI	2 (28.6%)	3 (33.3%)	
Cancer recurrence, *n* (%)	1 (14.3%)	6 (66.7%)	0.036
DFS, mean (months)	19.2 ± 2.5	12.7 ± 2.6	0.125
Follow Up, mean (months)	13.9 ± 7.1	17.6 ± 6.8	0.305

The variables were compared between the two groups using a Chi-square test. BMI: body mass index; M: male; F: female; DFS: disease-free survival.

**Table 3:** Chemotherapy protocols used in the study to assess the therapeutic responses of PDAC zPDXs. The drugs in the combinations and their concentrations are reported.

Chemotherapy Protocol	Drugs Combination	Concentration (mg/mL)
Gemcitabine	Gemcitabine	0.067
GEMOX	Gemcitabine	0.067
	Oxaliplatin	0.007
GEM/nab-P	Gemcitabine	0.067
	nab-Paclitaxel	0.008
FOLFOXIRI	5-Fluorouracil	0.216
	Folinic acid	0.013
	Oxaliplatin	0.006
	Irinotecan	0.011

**Table 4:** Definition of criteria used to define the zPDX response (adapted from WHO standard criteria).

Progressive Disease (PD)	Increase ≥ 25% in the relative stained area at 2 dpi
Stable Disease (SD)	Decrease or increase < 25% in the relative stained area at 2 dpi
Minor Response (MR)	Decrease ≥ 25% but < 50% in the relative stained area at 2 dpi
Partial Response (PR)	Decrease ≥ 50% but < 90% in the relative stained area at 2 dpi
Complete Response (CR)	Decrease ≥ 90% in the relative stained area at 2 dpi

## Data Availability

Dataset and metadata generated and/or analyzed during the current study are available from the corresponding author on request.

## Supplementary Materials

The following are available online at https://www.mdpi.com/article/10.3390/cancers13164131/s1, Figure S1: Means ad 95% CI of random effect in GEMOX, GEM, FOLFOXIRI and GEM/nab-P, Figure S2: PDAC zPDX error bars with 95% CI of fitted values estimated through LMM. Red dashed line is 0 on the log scale, Table S1: Fixed effects coefficients, their Standard Error (SE) and p-value estimated by LMM; Estimation of the radius; R packages.
