# Supplementary material for: Zebrafish Patient-Derived Xenografts Identify Chemo-Response in Pancreatic Ductal Adenocarcinoma Patients
Source: Cancers (Basel). 2021 Aug 17;13(16):4131. doi: 10.3390/cancers13164131 (PMC8394309; doi:10.3390/cancers13164131)
Supplement: Supplementary file 1 [file cancers-13-04131-s001.zip › cancers-1293619-supplementary.pdf]

cancers

MDPI

Supplementary Materials: Zebrafish Patient-Derived Xenografts Identify Chemo-Response in Pancreatic Ductal Adenocarcinoma Patients

Alice Usai, Gregorio Di Franco, Margherita Piccardi, Perla Cateni, Luca Emanuele Pollina, Caterina Vivaldi, Enrico Vasile, Niccola Funel, Matteo Palmeri, Luciana Dente, Alfredo Falcone, Dimitri Giunchi, Alessandro Massolo, Vittoria Raffa, Luca Morelli

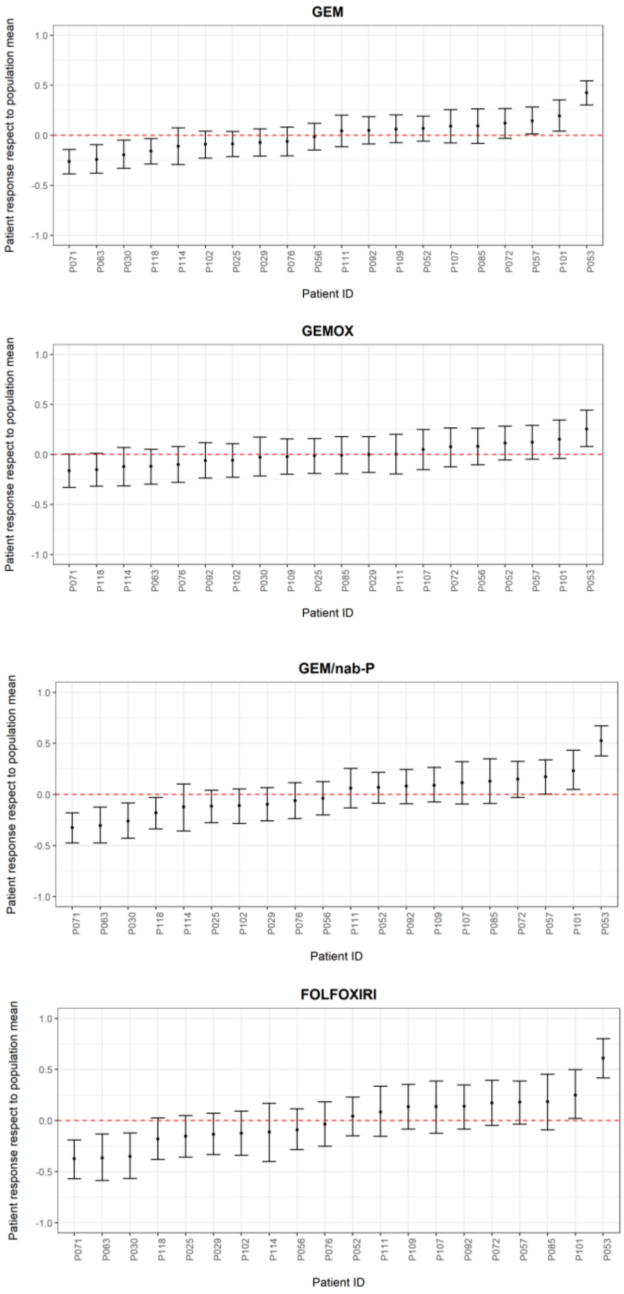

Figure S1. Means ad 95%CI of random effect in GEMOX, GEM, FOLFOXIRI and GEM/nab-P. Red dashed line represents the zebrafish embryos mean response to treatment. In x-axis is reported the patient ID and in y-axis the differences between zPDX and embryos mean responses.

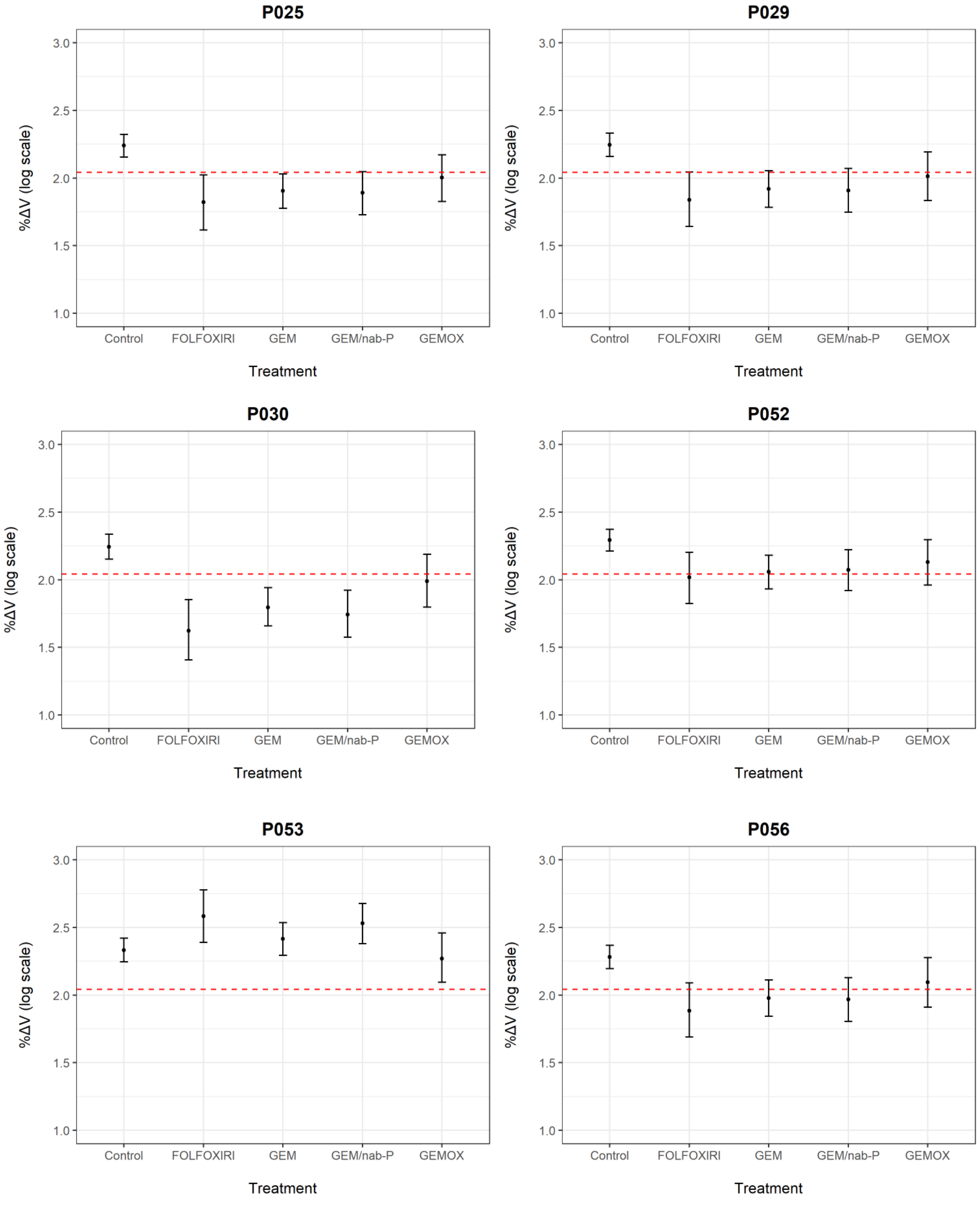
P025
P029
P030
P052
P053
P056
%ΔV (log scale)
3.0
2.5
2.0
1.5
1.0
Control
FOLFOXIRI
GEM
GEM/nab-P
GEMOX
Treatment

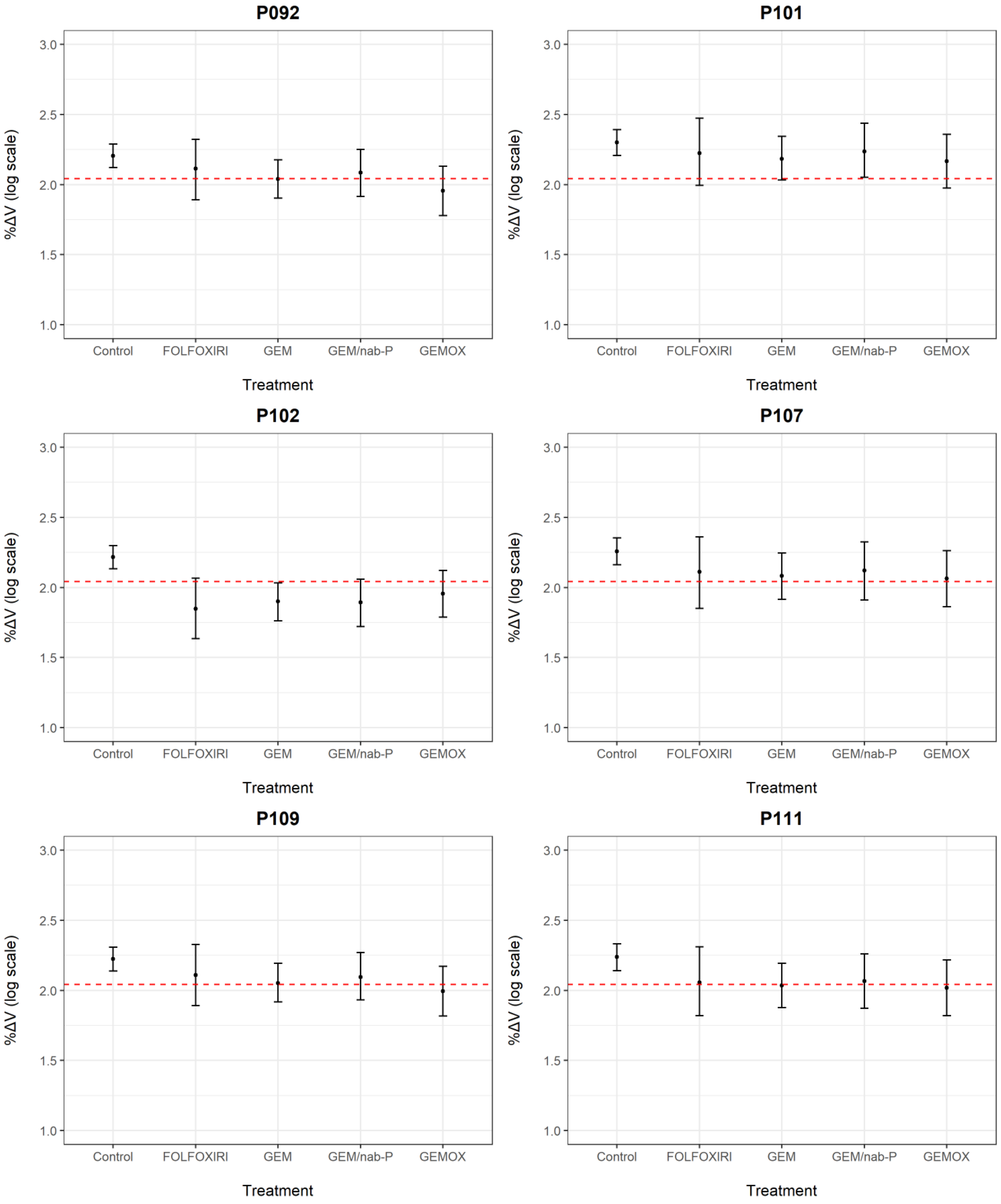
P092
P101
P102
P107
P109
P111
%ΔV (log scale)
1.0
1.5
2.0
2.5
3.0
Control
FOLFOXIRI
GEM
GEM/nab-P
GEMOX
Treatment

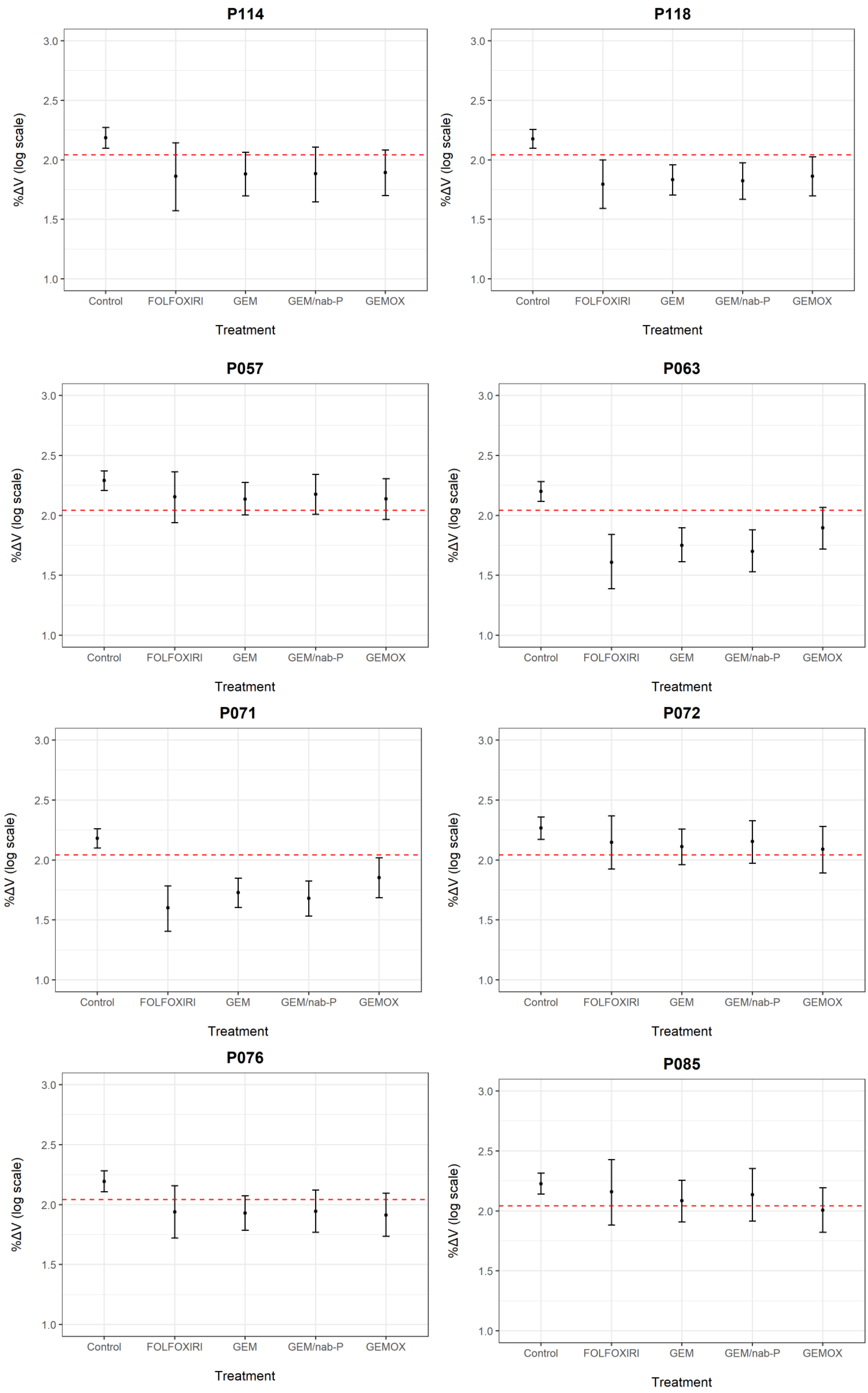
P114
P118
P057
P063
P071
P072
P076
P085
%ΔV (log scale)
3.0
2.5
2.0
1.5
1.0
Control
FOLFOXIRI
GEM
GEM/nab-P
GEMOX
Treatment

Figure S2. PDAC zPDX error bars with 95% CI of fitted values estimated through LMM. Red dashed line is 0 on the log scale.

Table S1. Fixed effects coefficients, their Standard Error (SE) and *p*-value estimated by LMM.

Treatment	Estimate	SE	*p*-Value
Control	2.24	0.04	<2.00×10-16
FOLFOXIRI	-0.27	0.08	2.40×10-3
GEM	-0.25	0.06	3.07×10-4
GEM/nab-P	-0.23	0.07	1.92×10-3
GEMOX	-0.22	0.06	2.16×10-4

Estimation of the radius

Starting from zPDX area measured at 1dpi and 2dpi, we estimated radius as:

$$r = \frac{\sqrt[2]{Area_{(1dpi\ or\ 2dpi)}}}{\pi}$$

R packages

1. *car* package:
John Fox and Sanford Weisberg (2019). An {R} Companion to Applied Regression. Third Edition. Thousand Oaks CA: Sage. URL: https://socialsciences.mcmaster.ca/jfox/Books/Companion/
2. *dplyr* package:
Hadley Wickham. Romain François. Lionel Henry and Kirill Müller (2020). dplyr: A Grammar of Data Manipulation. R package version 1.0.1. https://CRAN.R-project.org/package=dplyr
3. *ggplot2* package:
H. Wickham. ggplot2: Elegant Graphics for Data Analysis. Springer-Verlag New York. 2016.
4. *corrplot* package:
Taiyun Wei and Viliam Simko (2017). R package "corrplot": Visualization of a Correlation Matrix (Version 0.84). Available from https://github.com/taiyun/corrplot
5. *reshape2* package:
Hadley Wickham (2007). Reshaping Data with the reshape Package. Journal of Statistical Software. 21(12). 1-20. URL http://www.jstatsoft.org/v21/i12/.
6. *patchwork* package:
Thomas Lin Pedersen (2020). patchwork: The Composer of Plots. R package version 1.1.0. https://CRAN.R-project.org/package=patchwork
7. *readxl* package:
Hadley Wickham and Jennifer Bryan (2019). readxl: Read Excel Files. R package version 1.3.1. https://CRAN.R-project.org/package=readxl
8. *GGally* package:
Barret Schloerke. Di Cook. Joseph Larmarange. Francois Briatte. Moritz Marbach. Edwin Thoen. Amos Elberg and Jason Crowley (2020). GGally: Extension to 'ggplot2'. R package version 2.0.0. https://CRAN.R-project.org/package=GGally
9. *broom* package:
David Robinson. Alex Hayes and Simon Couch (2020). broom: Convert Statistical Objects into Tidy Tibbles. R package version 0.7.2. https://CRAN.R-project.org/package=broom
10. *parameters* package:
Lüdecke D. Ben-Shachar M. Patil I. Makowski D (2020). "parameters: Extracting. Computing and Exploring the Parameters of Statistical Models using R." _Journal of Open Source Software_. *5*(53). 2445. doi: 10.21105/joss.02445 (URL:https://doi.org/10.21105/joss.02445).
11. *effects* package:
John Fox and Sanford Weisberg (2019). An R Companion to Applied Regression. 3rd Edition. Thousand Oaks. CA https://socialsciences.mcmaster.ca/jfox/Books/Companion/index.html
For predictor effects or partial residuals also cite:
John Fox. Sanford Weisberg (2018). Visualizing Fit and Lack of Fit in Complex Regression Models with Predictor Effect Plots and Partial Residuals. Journal of Statistical Software. 87(9). 1-27. URL https://www.jstatsoft.org/article/view/v087i09.
For generalized linear models also cite:

John Fox (2003). Effect Displays in R for Generalised Linear Models. Journal of Statistical Software. 8(15). 1-27. URL https://www.jstatsoft.org/article/view/v008i15.

12. *ggeffect* package:
    Lüdecke D (2018). “ggeffects: Tidy Data Frames of Marginal Effects from Regression Models.” _Journal of Open Source Software_. *3*(26). 772. doi: 10.21105/joss.00772 (URL: https://doi.org/10.21105/joss.00772).
13. *emmeans* package: Russell Lenth (2020). emmeans: Estimated Marginal Means. aka Least-Squares Means. R package version 1.5.2-1. https://CRAN.R-project.org/package=emmeans
14. *performance* package: Lüdecke. Makowski. Waggoner & Patil (2020). Assessment of Regression Models Performance. CRAN. Available from https://easystats.github.io/performance/
15. *lme4* package: Douglas Bates. Martin Maechler. Ben Bolker. Steve Walker (2015). Fitting Linear Mixed-Effects Models Using lme4. Journal of Statistical Software. 67(1). 1-48.doi:10.18637/jss.v067.i01.
16. *arm* package: Andrew Gelman and Yu-Sung Su (2020). arm: Data Analysis Using Regression and Multilevel/Hierarchical Models. R package version 1.11-2. https://CRAN.R-project.org/package=arm
17. *lmerTest* package: Kuznetsova A. Brockhoff PB. Christensen RHB (2017). “lmerTest Package: Tests in Linear Mixed Effects Models.” _Journal of Statistical Software_. *82*(13). 1-26. doi: 10.18637/jss.v082.i13 (URL: https://doi.org/10.18637/jss.v082.i13)
18. *see* package: Lüdecke. Ben-Shachar. Waggoner & Makowski (2020). Visualisation Toolbox for 'easystats' and Extra Geoms. Themes and Color Palettes for 'ggplot2'. CRAN. Available from https://easystats.github.io/see/
19. *MuMIn* package: Kamil Barton (2020). MuMIn: Multi-Model Inference. R package version 1.43.17. https://CRAN.R-project.org/package=MuMIn
20. *factoextra* package: Alboukadel Kassambara and Fabian Mundt (2020). factoextra: Extract and Visualize the Results of Multivariate Data Analyses. R package version 1.0.7. https://CRAN.R-project.org/package=factoextra
21. *factoMineR* package: Sebastien Le. Julie Josse. Francois Husson (2008). FactoMineR: An R Package for Multivariate Analysis. Journal of Statistical Software. 25(1). 1-18. 10.18637/jss.v025.i01
22. *cluster* package: Maechler. M.. Rousseeuw. P.. Struyf. A.. Hubert. M.. Hornik. K.(2019). cluster: Cluster Analysis Basics and Extensions. R package version 2.1.0.
23. *stats* package: R Core Team (2020). R: A language and environment for statistical computing. R Foundation for Statistical Computing. Vienna. Austria. URL https://www.R-project.org/.
24. *merTools* package: Jared E. Knowles and Carl Frederick (2020). merTools: Tools for Analyzing Mixed Effect Regression Models. R package version 0.5.2. https://CRAN.R-project.org/package=merTools
25. *psych* package: Revelle. W. (2020) psych: Procedures for Personality and Psychological Research. Northwestern University. Evanston. Illinois. USA. https://CRAN.R-project.org/package=psych Version = 2.0.9..
26. *classInt* package: Roger Bivand (2020). classInt: Choose Univariate Class Intervals. R package version 0.4-3. https://CRAN.R-project.org/package=classInt
27. *dfidx* package: Yves Croissant (2020). dfidx: Indexed Data Frames. R package version 0.0-3. https://CRAN.R-project.org/package=dfidx
28. *forcats* package: Hadley Wickham (2020). forcats: Tools for Working with Categorical Variables (Factors). R package version 0.5.0. https://CRAN.R-project.org/package=forcats
